# Induction of TLR4/TLR2 Interaction and Heterodimer Formation by Low Endotoxic Atypical LPS

**DOI:** 10.3389/fimmu.2021.748303

**Published:** 2022-01-24

**Authors:** Sara Francisco, Jean-Marc Billod, Javier Merino, Carmen Punzón, Alicia Gallego, Alicia Arranz, Sonsoles Martin-Santamaria, Manuel Fresno

**Affiliations:** ^1^ Diomune S. L., Parque Científico de Madrid, Madrid, Spain; ^2^ Department of Molecular Biology, Centro de Biología Molecular “Severo Ochoa”, Consejo Superior de Investigaciones Cientificas (CSIC)-Universidad Autónoma de Madrid, Madrid, Spain; ^3^ Department of Structural Biology, Centro de Investigaciones Biologicas “Margarita Salas”, Consejo Superior de Investigaciones Cientificas (CSIC), Madrid, Spain

**Keywords:** Toll-like receptors, macrophages, innate immunity, atypical-LPS, cytokines

## Abstract

The Toll-like receptor 4 (TLR4)/myeloid differentiation protein-2 (MD-2) complex is considered the major receptor of the innate immune system to recognize lipopolysaccharides (LPSs). However, some atypical LPSs with different lipid A and core saccharide moiety structures and compositions than the well-studied enterobacterial LPSs can induce a TLR2-dependent response in innate immune cells. *Ochrobactrum intermedium*, an opportunistic pathogen, presents an atypical LPS. In this study, we found that *O. intermedium* LPS exhibits a weak inflammatory activity compared to *Escherichia coli* LPS and, more importantly, is a specific TLR4/TLR2 agonist, able to signal through both receptors. Molecular docking analysis of *O. intermedium* LPS predicts a favorable formation of a TLR2/TLR4/MD-2 heterodimer complex, which was experimentally confirmed by fluorescence resonance energy transfer (FRET) in cells. Interestingly, the core saccharide plays an important role in this interaction. This study reveals for the first time TLR4/TLR2 heterodimerization that is induced by atypical LPS and may help to escape from recognition by the innate immune system.

## Introduction

The innate immune system provides a first line of defense against a broad spectrum of pathogens. Macrophages, dendritic cells, and neutrophils contain a variety of pattern recognition receptors (PRRs) that sense the presence of pathogens (1). Upon binding to conserved pathogen-associated molecular patterns (PAMPs) (2), PRRs trigger a cascade of signals that induce the production of proinflammatory mediators determinant for pathogen killing and activation of the adaptive immune system.

The lipopolysaccharide (LPS) present in the Gram-negative cell wall is a well-described inducer of the innate immune response. The LPS structure comprises a lipid A composed of fatty acid (FA) chains linked to a disaccharide backbone, a core saccharide and the O-antigen (3). Toll-like receptor 4 (TLR4) complexed with myeloid differentiation protein-2 (MD-2) recognizes the lipid A portion of the LPS molecule. The *Escherichia coli* LPS is one of the most potent agonists of TLR4, containing a lipid A with six acyl chains, where five of them are buried inside the MD-2 pocket and the sixth chain is exposed to the MD-2 surface. The inner core of *E. coli* LPS consists of three units of 3-deoxy-d-manno-2-octulosonic acid (KDO I, II, III) and three units of heptosyl-2-keto-3-deoxy-octulosonate (Hep I, II, III) that establish hydrogen bonds with MD-2 and TLR4 but not with TLR4*. Thus, it is speculated that the core has a minor role in the immunological activity of LPS (4).

Several studies have highlighted that the biological activity of some LPSs is not restricted to TLR4. Indeed, *Leptospira interrogans*, *Legionella pneumophila*, and *Rhizobium* spp. LPSs induce TLR2-mediated inflammatory responses in immune cells (5–8). These atypical LPSs show a diaminoglucose disaccharide backbone, at least one very long FA chain (VLCFA) in the lipid A moiety and a core that differs in composition and charge compared with the enterobacterial-type LPS core. In addition, α-Proteobacteria pathogens like *Brucella abortus*, *Ochrobactrum anthropi*, and *Ochrobactrum intermedium* also express this type of LPS, sharing a similar lipid A with a VLCFA and a core composition that departs from the typical heptose sugar repetitions observed in *E. coli* core (9, 10).

The presence of atypical LPSs may be one of the properties associated with low virulence of stealth opportunistic pathogens. Most of the studies in this field demonstrated that for some LPSs, this reflects a poor agonistic or antagonistic activity for TLR4. For example, *Porphyromonas gingivalis* lipid A shows an antagonistic activity that enables the pathogen to evade TLR4-mediated bactericidal activity in macrophages, resulting in systemic inflammation (11). On the other hand, *Brucella* LPS is a poor TLR4 agonist and antigen-presenting cell activator (12–15), and its closely related species *O. anthropi* and *O. intermedium* are emerging as human opportunistic pathogens with mild virulence (16). Nevertheless, their immunomodulatory activity and the molecular basis of pathogen receptor involved in their interaction with the immune system are not fully understood.

In this study, we demonstrate that *O. intermedium* LPS induces a very low inflammatory response, which is dependent on TLR4 and TLR2 receptor interaction. Fluorescence resonance energy transfer (FRET) analysis demonstrates that this LPS favors unusual heterodimerization of TLR2 with TLR4 otherwise being independent molecules. Furthermore, docking studies of this LPS with a TLR2/TLR4/MD-2 computational model reveals the impact of the core saccharide in the low reactivity of this atypical LPS with the immune system.

## Materials and Methods

### Ethics Statement

This study was carried out in strict accordance with the European Commission legislation for the protection of animals used for scientific purposes (2010/63/EU). The protocol for the treatment of the animals was approved by the Comité de Ética de la Dirección General del Medio Ambiente de la Comunidad de Madrid, Spain (permits PROEX 21/14 and PROEX 148/15). Animals had unlimited access to food and water. They were euthanized in a CO_2_ chamber, and all efforts were made to minimize their suffering.

### Ligands

TLR4 ligand LPS from *E. coli* O111:B4 (Sigma) and TLR2/6 ligand FSL-1 (InvivoGen) were resuspended in sterile 1× PBS. *B. abortus* and *O. anthropi* LMG 3331 were obtained from Julian Velasco. *O. intermedium* LGM 3306 LPS was purified and characterized as previously described (17, 18). The purity of those compounds was assessed by mass spectrometry with a purity level higher than 98%.

### Cell Lines

The murine macrophage cell line J744 was cultured in RPMI 1640 medium (Gibco) (2 mM L-glutamine, antibiotics), supplemented with 5% FBS (Merck). Cells were cultured in 12-well plates and serum deprived for 16 h prior to ligand stimulation. HEK293T, HEK TLR2, and HEK TLR4/MD-2/CD14 were cultured in Dulbecco’s modified Eagle’s medium (DMEM; Gibco) (2 mM glutamine, 2 mM Aminoacids non-essential (AANE) 1% penicillin-streptomycin) with 5% fetal bovine serum (FBS) and incubated overnight before use.

### Isolation of Mouse Peritoneal Macrophages

C57BL/6 wild-type (WT) and TLR2 and TLR4 knockout (KO) mice were obtained from S. Akira and maintained in the animal facilities of the Centro de Biologia Molecular Severo Ochoa in Universidad Autonoma de Madrid. Thioglycolate-elicited peritoneal macrophages (PMs) were isolated from 6–8-week-old pathogen-free mice. Cells cultured in RPMI 1640-supplemented 2 mM L-glutamine, antibiotics (Gibco) and with 5% FBS and seeded into 12- or 6-well plates at a density of 1 × 10^6^ cells/well. Cells were allowed to adhere for 2 h, and then the medium was changed to remove non-adherent cells and incubated overnight before use.

### mRNA Isolation and RT-qPCR

Total cellular RNA was isolated using NZyol Reagent (NZYTech). cDNA was prepared by reverse transcription (GoTaq 2-Step RT-qPCR System, Promega) and amplified by PCR using SYBR^®^ Green PCR Master Mix and ABI Prism 7900HT sequence detection system (Applied Biosystems). Primers used for qPCR analysis of tumor necrosis factor (TNF)-α, interleukin (IL)-6, IL-10, and IL-12 are listed in **Supplementary Table S1**. The 2−^ΔΔCt^ method was applied to analyze the relative changes in expression profiling, and all quantifications were normalized to the housekeeping genes, Glyceraldehyde 3-phosphate dehydrogenase (GADPH) and RPL13A.

### ELISA

Cytokine concentration was determined for IL-10, TNF-α, IL-6, and IL2-p40 using DuoSet ELISA kit from R&D Systems according to the manufacturer’s protocol.

### Western Blot

Cells were lysed in ice-cold lysis buffer [50 mM Tris pH 7.5, 150 mM NaCl, 1% Triton X-100, 1 mM ethylenediaminetetraacetic acid (EDTA), 10% glycerol], phosphatase and protease inhibitors (Roche). Equal protein amount (20 µg) from each cell lysate was separated on sodium dodecyl sulfate (SDS) 10% polyacrylamide gel and transferred to a nitrocellulose membrane (Bio-Rad). Membranes were blocked with 3% bovine serum albumin (BSA) for 1 h and incubated with antibodies against IκBα (9242), p-p38 (Thr^180^/Tyr^182^) (9211), total p38 (9212), p-ERK1/2 (Thr^202^/Tyr^204^) (9101), and total ERK 1/2 (9102) from Cell Signaling and β-actin (sc-47778) from Santa Cruz Biotechnology. The membranes were then incubated with the respective horseradish peroxidase (HRP)-conjugated secondary antibody for 1 h and developed using enhanced chemiluminescence (ECL) substrate (BioRad).

### HEK-TLR-Expressing Cells NF-κB Reporter Assays

Stable immortalized HEK 293T, HEK 293-hTLR2/6, and HEK 293/hTLR4A-MD-2-CD14 cells (InvivoGen, San Diego, CA, USA) were plated at 3 × 10^6^ cells in 6-well plates growing at 37°C in DMEM culture medium supplemented with 5% FBS, 2 mM L-glutamine, 100 U/ml gentamycin, 0.01% pyruvate, and 0.4 mM non-essential amino acids. Then, 24 h later, cells were cotransfected with the pNF3ConA Luc [nuclear factor (NF)-κB] Firefly reporter construct and the thymidine kinase promoter-Renilla reporter plasmid (100:1 ratio) using Metafectene PRO (Biontex, Plannegg, Germany) (19). Transfection medium was changed after 24 h, and cells were seeded at 1.3 × 10^4^ cells per well in 96-well plates. Then, 24 h later, ligands were added. Activities of Firefly and Renilla luciferases were measured 24 h after using TwinLite Firefly and Renilla Luciferase Reporter Gene Assay System (PerkinElmer) in Fluo Star Optima (BMG) plate reader (three replicates per condition). All ratios were compared with the control condition (non-stimulated cells). We used FSL-1 (InvivoGen), TNF-α (InvivoGen), and LPS purified from *E. coli* (Sigma) and *O. intermedium* LPS.

### Molecular Docking

The full structure of *E. coli* LPS was extracted from the (hTLR4/MD-2/*E. coli*)_2_ complex retrieved from the Protein Data Bank (PDB ID 3FXI) (www.rcsb.org; last accessed July 12, 2021) (incluir la REF). The three-dimensional (3D) structure of *O. intermedium* LPS was constructed using PyMOL (20). *O. intermedium* LPS structure was then divided into fragments: Ocore (fragment containing the saccharide core of the LPS), O28 (fragment with the two FA Ocore chains containing 12 and 16 carbons and the third 28-carbon acyl chain attached to C16), O19 (fragment containing the two lipid chains with 14 and 18 carbons and the third 19-carbon lipid chain attached to C18), and lipid A. Cuts were introduced as hydrolysis of ether bonds, thus a hydrogen atom was added to the oxygen atom of the fragment. The structures went through a restrained minimization procedure with Maestro using the OPLS3 force field (21). The X-ray crystal structures of hTLR2/6 dimer (PDB ID 2Z7X) and hTLR4 (PDB ID 3FXI) were retrieved from the PDB. PyMOL was used to superimpose TLR2 to one monomer of the TLR4/TLR4 dimer. The final structure comprises TLR4/MD-2 complex dimerized with TLR2, the remaining atoms were deleted. The structures went through a minimization with Amber14 (22) under the ff14SB (23) to minimize the newly constructed TLR4/TLR2 interface.

Gasteiger charges were computed with AutoDockTools 1.5.6 (24) to ligands and receptors, and non-polar hydrogens of the receptors were merged. The structure of the receptors was kept rigid, whereas all ligands were set to be partially flexible by providing rotational freedom to some appropriately selected dihedral angles. The docking calculations were performed with Autodock Vina (25). Each ligand was docked into different regions of hTLR2 monomer and hTLR2/TLR4/MD-2 heterodimer complex by placing different boxes at different regions. For all the docking boxes, the point spacing was set as 1 Å. For hTLR2, a docking was performed with the box covering the TLR2 pocket with a center placed equidistant to the center of mass of residues Phe284, Leu282, and Asn274 and a box size of 40, 36, 30 (X, Y, Z). For hTLR2/TLR4/MD-2 heterodimer, a docking was performed in the region behind hTLR2 containing the N-terminal and central subdomains, and the center of the box was located equidistant to the center of mass of residues Arg321, His318, and Asn290 with a size of 37, 50, 50 (X, Y, Z). Another box was set to cover the interface of TLR2 and TLR4 receptor centered equidistant to the center of mass of Lys324 (TLR4), Tyr376 (TLR2), and Asn379 (TLR2) and of size 37, 50, 50 (X, Y, Z). The docking was also performed inside the MD-2 pocket, setting a box where the center of coordinates was equidistant to the center of mass of Phe119 (TLR4), Ile52 (MD-2), and Ser57 (MD-2) (X, Y, Z) and size 36, 38, 50 (X, Y, Z). A docking covering the MD-2 pocket of the hTLR4/MD-2 homodimer was also performed. A box of 60 Å in size was defined and centered equidistant to the center of mass of residues Arg90 (MD-2), Arg96 (MD-2), and Arg264 (TLR4). The determination of the best result from each docking was based on the predicted binding energy and the mode of interaction of the ligands. Docking poses were analyzed, and structural images were generated in PyMol.

### Cell Transient Transfection and Fluorescence Resonance Energy Transfer Imaging

HEK293T cells were seeded in 6-well plates (3 × 10^5^ cells/well) and incubated overnight in DMEM with 5% FBS without antibiotics. On the next day, cells were transiently transfected with 0.5 µg of plasmid mixture of pcDNA3-hTLR4-YFP and pcDNA3-hTLR2-CFP (Addgene) or transfected with each plasmid (molar ratio 1:1) using Metafectene Pro (Biontex), according to the manufacturer’s protocol. Cells were also transfected with the plasmid pCMV-ECFP-EYFP (Addgene) that expresses the tandem CFP : YFP construct, which served as a positive control for FRET. A day after transfection, cells were cultured in 8-well glass bottom chambers and incubated overnight in phenol red-free DMEM with 5% FBS and 25 mM 4-(2-hydroxyethyl)-1-piperazineethanesulfonic acid (HEPES). The following day, cells were stimulated with *O. intermedium* LPS for 30 min. FRET between TLR2 and TLR4 proteins was calculated by measuring sensitized emission fluorescence of CFP-YFP pair using NIS Elements 4.40 software on the Nikon Eclipse Ti-E confocal microscope. FRET image acquisition and processing details are provided in the **Supplementary Material**.

### Statistics

Analysis was performed using GraphPad Prism 5 software. Quantitative results are expressed as means ± SEM or mean ± SD. Statistical analysis between two groups was performed using two-tailed unpaired Student’s t-test. Two or more groups were compared with one-way ANOVA followed by Bonferroni multiple comparisons. A p-value <0.05 was considered statistically significant.

## Results

### Atypical Lipopolysaccharide From *Ochrobactrum intermedium* Triggers Lower Pro-Inflammatory Responses Than *Escherichia coli* Lipopolysaccharide

**Figure 1:**
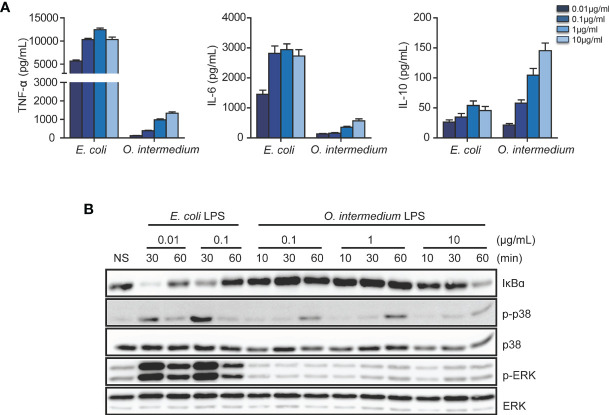
Lipopolysaccharide (LPS) from *O. intermedium* induces lower proinflammatory cytokine levels compared to the *E*. *coli* LPS. **(A)** Macrophage cell line J774 was stimulated with *O. intermedium* LPS or *E*. *coli* LPS at a concentration of 0.01, 0.1, 1, and 10 µg/ml for 24 h. Cytokine secretion was assayed from macrophage supernatants, and data are expressed as mean ± SD of three independent experiments. **(B)** Wild-type (WT) peritoneal macrophages were treated with 2 doses of *E*. *coli* LPS or 3 distinct doses of *O. intermedium* LPS for different indicated time points. Nuclear factor (NF)-κB activation was determined by degradation of IκBα and mitogen-activated protein kinase (MAPK) [p38 and extracelllular regulated kinase (ERK)] activation by phosphorylation in Western blot. The data are representative of three independent experiments.

We compared the production of inflammatory cytokines in J774 macrophages treated with *E. coli* and *O. intermedium* LPS at different doses. The cytokine induction was dose-dependent for both LPSs, but reaching a plateau for *E. coli* LPS at 0.1 μg/ml, whereas *O. intermedium* LPS required much higher doses. However, levels of proinflammatory cytokines TNF-α and IL-6 induced by *O. intermedium* were much lower than those induced by *E. coli* LPS, even at 100-fold higher doses (**Figure 1A**) . IL-12 production was also induced at lower levels by *O. intermedium*. However, in contrast, *O. intermedium* triggered higher IL-10 expression levels compared to *E. coli* LPS. In addition, NF-κB and mitogen-activated protein kinase (MAPK) activation induced by O. intermedium LPS increases in a dose-dependent manner; however, the response was always much weaker compared to that of E. coli LPS (**Figure 1B**). Moreover, these results indicate that the atypical LPS from *O. intermedium* is a weaker proinflammatory inducer compared to enterobacterial LPS.

### *O. intermedium* Lipopolysaccharide Induces an Inflammatory Response Mediated by TLR4 and TLR2 Receptors

**Figure 2:**
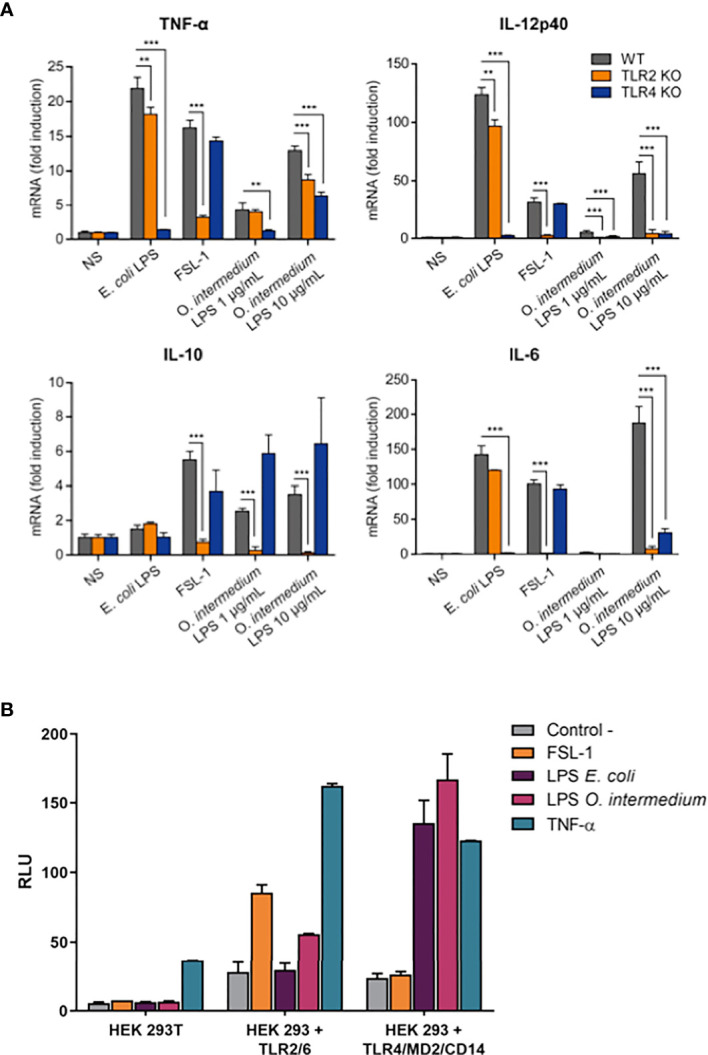
*O. intermedium* lipopolysaccharide (LPS) gives a weak inflammatory response mediated by Toll-like receptor (TLR)2 and TLR4. **(A)** Wild-type (WT), TLR2 knockout (KO), and TLR4 KO peritoneal macrophages were left unstimulated (NS) or were stimulated with *E*. *coli* LPS (100 ng/ml), FSL-1 (100 ng/ml), and *O. intermedium* LPS (1 and 10 µg/ml) for 24 h. Cytokine mRNA levels were measured by qPCR. Data ate representative of two independent experiments and expressed as the mean ± SEM. **p < 0.01, and ***p < 0.001. **(B)** HEK 293T, HEK 293-hTLR2/6, and HEK 293/hTLR4A-MD-2-CD14 cells were cotransfected with the pNF3ConA Luc (NF-κB) firefly reporter construct and the thymidine kinase promoter-Renilla reporter plasmid and stimulated with FSL-1 (100 ng/ml), TNF (100 ng/ml), or LPS purified from *E*. *coli* (100 ng/ml) and *O. intermedium* LPS (10 μg/ml). Activity of Firefly and Renilla luciferases were measured 24 h after. RLU, relative luciferase light units.

Atypical LPSs have been proposed to induce inflammatory responses through TLR2 rather than TLR4 (5, 6). Therefore, we investigated the TLR4 and TLR2 dependency for O. intermedium LPS-induced inflammatory response. For this, we first tested their stimulatory effects on WT and TLR2 and TLR4 KO peritoneal macrophages with two different doses of this LPS and compared with two controls: E. coli LPS (TLR4 ligand) and FSL-1 (TLR2 ligand). WT macrophages stimulated with O. intermedium LPS showed a weaker induction of cytokines compared to E. coli LPS. O. intermedium LPS only achieved cytokine levels similar to E. coli LPS at a 100-fold dose of 10 μg/ml (**Figure 2A**). Moreover, O. intermedium LPS showed dependency on TLR4 and TLR2 to induce cytokine production. IL-12, IL-6, and TNF-α were dependent on TLR2 and mostly on TLR4 signaling, whereas IL-10 induction was mostly TLR2-dependent. Next, both LPSs were tested in HEK transfected cells expressing TLR2/TLR6 or TLR4/CD14/MD-2 using NF-κB reporter assays. As expected, *E. coli* LPS only signals through TLR4, whereas we found that O. intermedium LPS could signal via TLR2 or TLR4 (**Figure 2B**). Nonetheless, as in previous proinflammatory assays, O. intermedium LPS was 100-fold less active than E. coli LPS in TLR4/CD14/MD-2 HEK transfected cells (**Supplementary Figure S1**).

Moreover, we found that co-activation of macrophages by E. coli LPS, a specificTLR4 ligand, plus specific TLR2 ligands as Pam3CSK4 or FSL-1 induces an additive effect on NF-κB and p38 signaling pathways, observed by an earlier p38 phosphorylation and IκBα degradation (**Supplementary Figure S2**). The same effect on NF-κB activation was observed when TLR2 ligands were combined with O. intermedium LPS (**Supplementary Figure S3**). This pattern was not observed with O. intermedium LPS, therefore supporting that this LPS is free of TLR2 contaminants that may be responsible for TLR2 signal. Overall, and more importantly, these results imply that *O. intermedium* LPS is a TLR4/TLR2-dependent agonist.

### Docking of *O. intermedium* Lipopolysaccharide in hTLR4/MD-2 Homodimer

**Figure 3:**
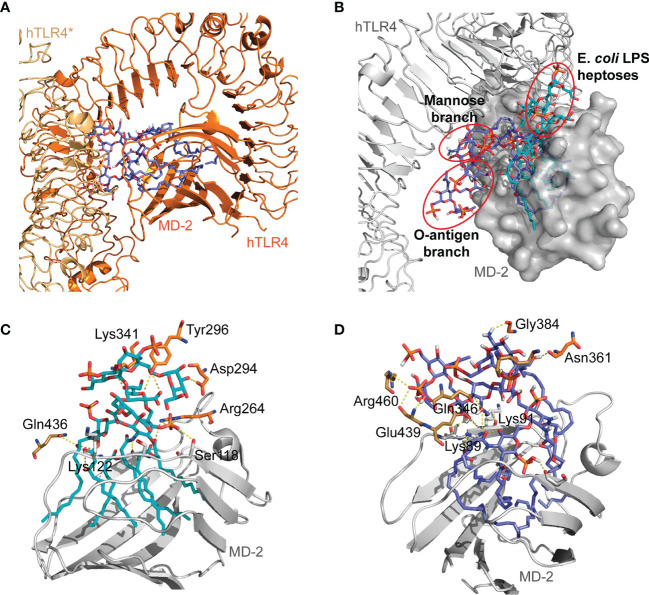
General view of *O. intermedium* lipopolysaccharide (LPS) and (*E*) *coli* LPS docked in hTLR4/MD-2 dimer. **(A)** Full *O. intermedium* LPS (in purple) conformation with the core diving toward TLR4*–TLR4 interface. **(B)** *E*. *coli* LPS pose (in cyan) superimposed with *O. intermedium* LPS pose (in purple) in hTLR4/MD-2 dimer (hTLR4 and MD-2 are in gray, and hTLR4/MD-2* was omitted for clarity). The polar interactions established between **(C)** *E*. *coli* LPS and **(D)** *O. intermedium* LPS with TLR4* (brown), TLR4 (in orange), and MD-2 (in gray) residues are presented. (An asterisk distinguishes the second TLR4/MD-2 monomer).

*E. coli* LPS recognition by hTLR4/MD-2 complex was previously described (4) where the inner core composed of KDO I, KDO II, and heptoses established important interactions with MD-2 (e.g., Ser118, Lys122) and TLR4 residues (e.g., Lys341, Tyr296, Asp294). Therefore, *O. intermedium* LPS was docked in hTLR4/MD-2 homodimer and superimposed with the *E. coli* LPS to compare possible binding orientations and the interactions of both LPSs with the two TLR4 monomers. The obtained binding poses showed that *O. intermedium* LPS FA chains were buried in the MD-2 pocket; however, the core saccharide dove toward TLR4*–TLR4 interface (**Figure 3A**). This is more evident when the docked pose of *O. intermedium* LPS and *E. coli* LPS are superimposed in the hTLR4/MD-2 homodimer (**Figure 3B**). *E. coli* LPS core established determinant interactions with TLR4 residues (e.g., Tyr296, Asp294, and Arg264) as well as with TLR4 (Gln436) (**Figure 3C**), whereas these interactions were not detected in the case of *O. intermedium* LPS (**Figure 3D**). These results indicate that, in contrast to *E. coli* LPS, *O. intermedium* LPS does not favor the formation of stable TLR4 homodimers.

### Docking of *O. intermedium* Lipopolysaccharide Binding in the Model hTLR2/TLR4-MD-2 Heterodimer

Given that *O. intermedium* LPS is a TLR4 and TLR2-dependent agonist, the ability of this LPS to bind to a hypothetical heterodimer formed by the human TLR2 and TLR4-MD-2 receptors was explored by a molecular docking approach. First, the 3D structure of the hypothetical hTLR2/TLR4-MD-2 heterodimer model was constructed (see **Materials and Methods** and **Supplementary Figure S4**) and was subsequently used to perform docking calculations with *E. coli* and *O. intermedium* LPSs. The *O. intermedium* LPS was also divided into fragments for specific molecular dockings.

*E. coli* LPS accommodates five of its six lipid A chains inside the MD-2 pocket, and the remaining chain is at the surface of MD-2, in the TLR4/MD-2 homodimer (4). Therefore, the ability of *O. intermedium* LPS fragments (O19, OV28, and Ocore) to bind to the MD-2 pocket in the hTLR2/TLR4/MD-2 complex was investigated by docking calculations. Favorable poses for these fragments were found, where the two lipid A fragments showed their three FA chains buried in the MD-2 pocket and the core was near the entrance of MD-2 (**Supplementary Figure S5**). As for the interactions, the glucosamine phosphate group was interacting with the NH_2_ groups of Arg264, which is the same residue that interacts with the glucosamine of the *E. coli* lipid A in the hTLR4/MD-2 X-ray crystal structure (PDBI ID 3FXI).

**Figure 4:**
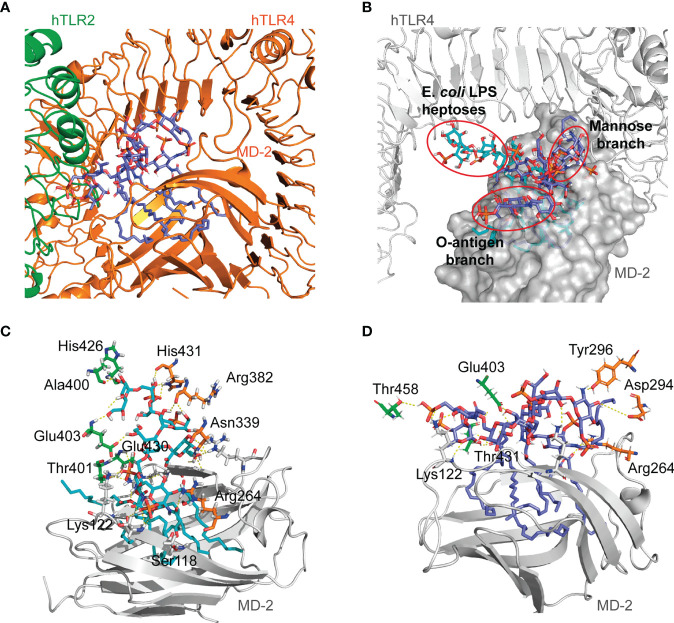
General view of *O. intermedium* lipopolysaccharide (LPS) and *E*. *coli* LPS docked in hTLR2/TLR4/MD-2 heterodimer. **(A)** *O. intermedium* LPS (in cyan) conformation with one fatty acid (FA) chain protruding out of the MD-2 pocket. **(B)** *E. coli* LPS docked pose (in cyan) was superimposed with *O. intermedium* LPS pose (in purple) in hTLR2/TLR4/MD-2 complex (hTLR4 and MD-2 are in gray, and hTLR2 was omitted for clarity). The polar interactions established between **(C)** *E*. *coli* LPS and **(D)** *O. intermedium* LPS with TLR2 (green), TLR4 (in orange), and MD-2 (on gray) residues are presented. (TLR4 and MD-2 are shown in orange, and TLR2 is in green).

In addition, full *O. intermedium* LPS was docked in the hTLR2/TLR4/MD-2 heterodimer to explore possible theoretical binding modes. The obtained binding poses had five FA chains of the LPS buried in the MD-2 pocket and the remaining sixth C12 chain placed on its surface (**Figure 4A**). In this case, both LPSs had their lipid A part accommodated in the MD-2 pocket. However, *E. coli* LPS core was diving toward the TLR2–TLR4 interface (**Figure 4B**). The interactions of the *E. coli* LPS core with the TLR4 residues described in the hTLR4/MD-2 dimer (Tyr296, Lys341, or Asp294) are not observed in the case of the hTLR2/TLR4/MD-2 heterodimer (**Figure 4C**). The TLR4 residues interacting with *E. coli* LPS core are closer to the TLR4 and TLR2 interface. This suggests that *E. coli* LPS does not favor the proximity of TLR4 and TLR2 receptors. On the other hand, *O. intermedium* LPS core maintains interactions with TLR4 residues such as Tyr296 and Arg264, as well as with TLR2 residues far from the interface region (**Figure 4D**). This implies that *O. intermedium* LPS accommodates in the hTLR2/TLR4/MD-2 complex in such a way that could favor the dimerization of both receptors. The *O. intermedium* LPS poses obtained from the docking calculations performed on the hTLR2/TLR4/MD-2 heterodimer and on the hTLR4/MD-2 homodimer hint that the core composition and/or structure of this LPS might favor the formation of an hTLR2/TLR4 complex rather than a TLR4 homodimer.

The ability of *O. intermedium* LPS core to bind in the hTLR2–hTLR4 interface region was also investigated. In this docking, more diversity in the binding poses was observed, where most of the poses were near the entrance of the MD-2 pocket and some poses featured the ligand in the upper region of the TLR2–TLR4 interface and toward the central domain of TLR2 (**Supplementary Figure S6A**).

The position of the *O. intermedium* LPS core in the hTLR2 interface (region spanning from the central domain, from Leu151 to Arg337, to the C-terminal domain, from Val338 to Iso506) shows interactions of the phosphate and hydroxyl groups with the backbone of TLR2, including residues His398, Ser425, Lys422, Gln396, Lys347, Asn345, Asp286, and Asn257 (**Supplementary Figure S6B**). Moreover, hydrogen bonds established with TLR4 residues Ser415, Gly389, and Lys388 were observed as well. The saccharide core from *E. coli* LPS was also docked in this region, suggesting that the *E. coli* core can theoretically interact with the TLR2 C-terminal domain (**Supplementary Figure S5C**) in an energetically favorable manner. The core also established hydrogen bonds with TLR4 Ser415, Lys388 similarly to *O. intermedium* LPS core; however, it showed fewer hydrogen interactions with TLR2 residues (His398, Gln396, and Asn345). Our calculations predict that the *O. intermedium* LPS core presents binding poses with more interactions with TLR2 residues located in the central and C-terminal domains. Therefore, *O. intermedium* LPS core seems better suited to establish interactions at the TLR2 interface compared to *E. coli* LPS core.

### Docking of *O. intermedium* Lipopolysaccharide Binding in the Model hTLR2 Monomer

**Figure 5:**
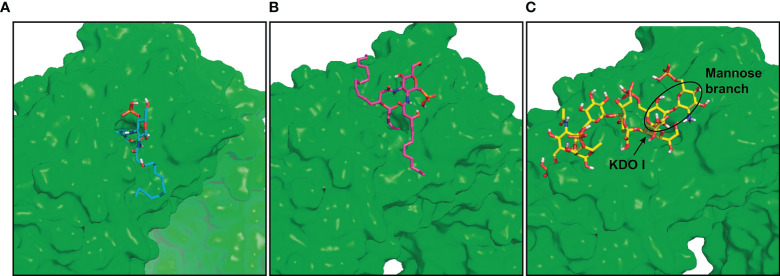
*O. intermedium* lipopolysaccharide (LPS) fragments docking in the hTLR2 pocket. Docking of the fragments **(A)** O19 (cyan), **(B)** O28 (magenta), and **(C)** Ocore (yellow) in the hTLR2 pocket (TLR2 surface is represented in green for easier visualization of the pocket).

TLR2 is described to associate with TLR1 or TLR6 receptors, and this is required for recognition of the tri- and diacylated lipopeptides, respectively (26–29). TLR2 contains an internal hydrophobic pocket where it accommodates two lipid chains from TLR2-specific lipopeptides (30). Since TLR2 can hypothetically interact with TLR4 by *O. intermedium* LPS, docking studies were carried out using this LPS fragment in a region that covers the TLR2 pocket. Fragments O19, O28, and Ocore were docked in the TLR2 pocket of the hTLR2 monomer model. The O19 fragment poses showed the two FA chains C19 and C18 buried in the TLR2 pocket and the C14 chain was displayed in the solvent (**Figure 5A**). On the other hand, the O28 fragment that comprises the large 28C chain was fully accommodated inside the pocket, whereas C12 and C16 chains protruded out of the TLR2 pocket (**Figure 5B**). Regarding the Ocore, the obtained docked poses were interacting with TLR2, near its pocket (**Figure 5C**). These results indicate that the lipid A component of *O. intermedium* LPS is theoretically able to interact with the hydrophobic TLR2 pocket, comprising a maximum of two acyl chains.

### *O. intermedium* Lipopolysaccharide Induces hTLR4 and hTLR2 Heterodimerization

**Figure 6:**
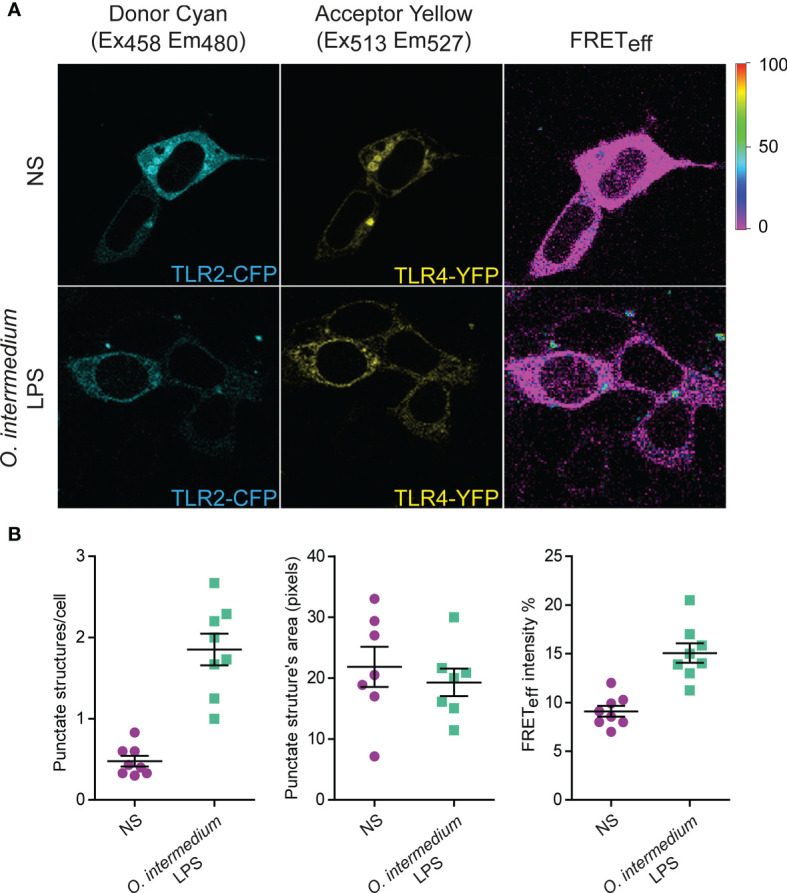
*O intermedium* lipopolysaccharide (LPS) induces hTLR4/hTLR2 heterodimerization. **(A)** HEK293T cells were transiently transfected with the plasmids hTLR4-YFP (acceptor) and hTLR2-CFP (donor). After transfection, cells were left unstimulated (NS) or were stimulated with *O. intermedium* LPS (1 µg/ml) for 10 min. Fluorescence resonance energy transfer (FRET) between YFP and CFP was measured in *in vivo* cells by sensitized emission fluorescence. Corrected FRET images (FRET_eff_) are displayed, and FRET efficiency is shown as a color-coded scale of values between 0% and 100%. **(B)** Quantification of FRET-positive structures per total cell number as well as the area and mean fluorescence intensity of each punctate structure. The results are shown as mean ± SEM of two independent experiments (p < 0.001 between treated and untreated cells in punctuate structures/cell and FRET efficiency).

As receptor dimerization appears to be required for PAMP recognition and TLR activation, FRET was performed to evaluate the intermolecular distance between TLR4 and TLR2 in the presence of *O. intermedium* LPS. HEK293T cells were transiently transfected with hTLR4-YFP (acceptor) and hTLR2-CFP (donor) constructs and imaged *in vivo*. Unstimulated cotransfected cells showed a small FRET signal, with minute punctate-like structures present in the cell membrane and cytoplasm. After stimulation with *O. intermedium* LPS, a significant FRET signal was observed by the presence of more punctate structures and with higher fluorescence intensity (**Figure 6A**). A tandem vector construct of CFP and YFP was used as a positive control, with a FRET efficiency of around 40% (data not shown). The size of these punctate structures however did not change upon cell stimulation (**Figure 6B**). This effect was mainly seen at high *O. intermedium* LPS (not shown) in agreement with the biochemical signaling data shown above. These results demonstrate that in resting cells, TLR4 and TLR2 receptors are not close enough. However, *O. intermedium* LPS stimulation promotes a closer interaction between TLR4 and TLR2 receptors that likely enables the activation of downstream intracellular signaling events. Thus, these data indicate that LPS from *O. intermedium* can induce the rearrangement of both TLR2 and TLR4 receptors favoring their interaction.

## Discussion

TLR4 is considered the major receptor involved in the recognition of all LPSs. However, this paradigm has been questioned and debated. Despite a general structure consisting of lipid A, core, and O-saccharide, LPS from different bacteria can present different compositions in those 3 parts. Those differences in LPS structure may affect their immunomodulatory activity. Thus, *Brucella* spp. and *O. anthropi* LPS are weaker proinflammatory activators than enterobacterial LPS due to lower affinity for TLR4 (12–14, 31). However, other weak proinflammatory agonist LPSs as those from *Legionella pneumophila*, *Rhizobium* species, or *P. gingivalis* were found to require TLR2 rather than TLR4 to elicit innate immune responses (6–8, 32). Some studies ascribed this effect to the contamination of purified LPS by lipoproteins that bind to TLR2. Nevertheless, purity may not be the full explanation, since those abovementioned LPSs present a structure and composition very different from the enterobacterial LPS, the archetypical ligand of TLR4. A common feature of some atypical LPSs is the presence of VLCFAs in the lipid A that reduces its recognition and a core saccharide and O-antigen with distinct sugars, importantly, with different net charge compared with the classical *E. coli* LPS. The different structures of *O. intermedium* and *E. coli* LPS are shown in **Supplementary Figure S7**. Members of α-Proteobacteria such as *Brucella* and *Ochrobactrum* express these atypical LPSs, and their peculiar structure may confer to these bacteria a stealthy strategy for immune system recognition (31, 33). However, those results have indicated that *Brucella* and *O. anthropi* LPSs only bind to TLR4 receptor.

The present study provides clear evidence that atypical *O. intermedium* LPS induces a weaker inflammatory response compared to *E. coli* LPS, mediated not only by TLR4 but also by TLR2; *O. intermedium* LPS lipid A is identical to *Brucella* spp. and *O. anthropi* LPSs that may explain its similar lower affinity for TLR4, resulting in a much weaker signal through TLR4 alone (12–14, 31).

In contrast to proinflammatory activation, anti-inflammatory IL-10 production is higher by *O. intermedium* LPS than by *E. coli* LPS. This supports that *O. intermedium* LPS has some affinity for TLR2, since pure TLR2 ligands induce higher levels of IL-10 compared with TLR4 ligands (34). Contamination of *O. intermedium* LPS with another TLR2 ligand is unlikely to be the explanation due to the following reasons: a) The effect observed with this contaminated LPS should have shown a characteristic of TLR2 signaling: an early NF-κB and MAPK activation (34). This was never observed even at 10 µg/ml of *O. intermedium* LPS (**Figure 1**); b) Mixing *O. intermedium* LPS with pure TLR2/1 or TLR2/6 ligands, at very low concentrations, results in a much faster and stronger NF-κB activation than with *O. intermedium* LPS alone (see **Supplementary Figure S3**).

More importantly, we provide direct physical evidence of TLR4-TLR2 dimerization upon *O. intermedium* LPS binding. Our molecular docking analysis elucidates the predictive interaction of *O. intermedium* LPS with hTLR4/MD-2 homodimers, showing its displacement from the MD-2 pocket, the core saccharide diving toward the interface of the complex, and loss of determinant interactions with TLR4 and TLR4 residues. On the other hand, the docking of this LPS in a putative hTLR2/TLR4/MD-2 heterodimer demonstrated that the lipid A accommodates in MD-2 pocket, the core side branch establishes determinant interactions with TLR4, and the O-antigen branch of the core seems to be a determinant for the interaction with TLR2. Therefore, *O. intermedium* LPS is likely to favor the formation of hTLR2/TLR4/MD-2 heterodimers rather than hTLR4/MD-2 homodimers. More importantly, our FRET results demonstrate that *O. intermedium* LPS induces TLR2 and TLR4 interactions as suggested by our docking studies. The weak contacts between toll-interleukin-1 receptor (TIR) domains of the dimers may impair the full recruitment of downstream adaptors by TLR2 and TLR4, leading to a poor activation of signaling cascades. This could explain the very weak proinflammatory agonist activity observed for *O. intermedium* LPS, requiring much higher doses to achieve similar activations than enterobacterial LPS. Previous studies have only demonstrated cytoplasmic TLR2-TLR4 binding using enzyme complementation assays (35) and by co-immunoprecipitation in cells from renal tubules (36).

Importantly, our study also suggests for the first time the importance of the core of the LPS for recognition by TLR molecules. In this regard, a previous study has found that besides the VLCFA, the core saccharide of *B. abortus* LPS hampers its MD-2 recognition (37). Moreover, *O. anthropi* LPS with a similar lipid A as *Brucella* has a different core that did not hamper MD-2 recognition activity being more proinflammatory, although not as much as enterobacterial LPS (31). *O. intermedium* core has a higher positive net charge than *E. coli* LPS core that may affect its interaction with TLRs favoring TLR4/TLR2 dimerization.

Many bacteria are properly recognized by the innate immune system, although others, as some α-Proteobacteria as *Brucella* and *Ochrobactrum* spp. are not easily detected, converting them into stealthy pathogens. Several studies have analyzed the role of *Brucella* LPS and its recognition by TLR4 in this [see (13) for a review]. Interestingly, *O. intermedium* core is different from *Brucella* spp. and *O. anthropi* cores that may explain some differences in their immune activation activity. Taken together, those data indicate that core structures together with lipid A FA composition of atypical LPSs may alter its interaction with TLR4/MD2 but also affect TLR4 dimerization. Likely, the 3D structure generated by core and lipid A may be very important in avoiding proper TLR recognition by some stealthy pathogens. Thus, LPS from very closely related species as *Ochrobactrum* and *Brucella* may avoid full innate immune recognition by several different mechanisms related to TLR recognition, such as hampering MD2 as *Brucella* or avoiding TLR4 heterodimerization and favoring TLR2/TLR4 heterodimerization as *O. intermedium.*

Another important aspect to consider is the coexistence of different species, i.e., LPS moieties together with the full LPS in aqueous solvents. Portions of lipid A or the core saccharide can be present as well. Considering this, *O. intermedium* lipid A fragments and the core were docked in the interface of TLR2 heterodimerized with TLR4/MD-2. Our results show that the *O. intermedium* core distributes in the TLR2 interface, whereas up to two FA chains of lipid A can be accommodated in the TLR2 pocket. Therefore, we propose a model consisting in a first dimerization step of TLR2 and TLR4/MD-2 monomers induced by full *O. intermedium* LPS binding in the MD-2 pocket, and consequently, LPS moieties can bind to TLR2 and likely contribute for the final dimerization process of TLR2 with a preassembled TLR4/MD-2 complex.

In summary, our findings have clearly pointed out for the first time the formation of TLR2/TLR4/MD-2 heterodimers in response to a particular atypical LPS, as well as the important undescribed role of the core structure in this interaction with TLRs besides the well-known lipid A from *E. coli*. Further molecular and biological studies are necessary to elucidate if LPS cores and O-antigen may contribute to the lower virulence of opportunistic pathogens. This is also relevant for the development of vaccine adjuvants and more efficient immunomodulators.

## Data Availability Statement

The original contributions presented in the study are included in the article/**Supplementary Material**. Further inquiries can be directed to the corresponding author.

## Ethics Statement

The animal study was reviewed and approved by Comité de Ética de la Dirección General del Medio Ambiente de la Comunidad de Madrid, Spain (permits PROEX 21/14 and PROEX 148/15).

## Author Contributions

SF, J-MB, AA, SM-S, and MF designed the different experiments. SF, J-MB, CP, JM, and AG performed the experiments. SF, J-MB, SM-S, and MF wrote the article. All authors contributed to the article and approved the submitted version.

## Funding

This research was funded by grants from the European Union “Host–microbe interactions in health and disease. Interface with the immune system”. HOMIN-317057-FP7-PEOPLE-2012-ITN to Diomune and Spanish MINECO (SAF2016-75988-R to MF and CTQ2017-88353-R to SM-S), Instituto de saludCarlos III (RD16/0027/0006), Comunidad de Madrid (S2017/BMD-3671. INFLAMUNE-CM) to MF, H2020-MSC-ETN-642157 project TOLLerant to SM-S and MF, and institutional grants from “Fundación Ramón Areces” and “Banco de Santander” to CBMSO.

## Conflict of Interest

Authors CP, SF and JM were employed by company Diomune SL. Author MF was an advisor of company Diomune SL.

The remaining authors declare that the research was conducted in the absence of any commercial or financial relationships that could be construed as a potential conflict of interest.

## Publisher’s Note

All claims expressed in this article are solely those of the authors and do not necessarily represent those of their affiliated organizations, or those of the publisher, the editors and the reviewers. Any product that may be evaluated in this article, or claim that may be made by its manufacturer, is not guaranteed or endorsed by the publisher.
