# Supplementary material for: Induction of TLR4/TLR2 Interaction and Heterodimer Formation by Low Endotoxic Atypical LPS
Source: Front Immunol. 2022 Jan 24;12:748303. doi: 10.3389/fimmu.2021.748303 (PMC8818788; doi:10.3389/fimmu.2021.748303)
Supplement: Supplementary file 1 [file DataSheet_1.docx]

**Supplementary information**

Induction of TLR4/TLR2 interaction and heterodimer formation by low endotoxic atypical LPS

Authors: Sara Francisco^1,2^, Jean-Marc Billod^3^, Carmen Punzón^1^, Alicia Arranz^2^*, Javier Merino^2^, Alicia Gallego^2^, Sonsoles Santamaria^3^ and Manuel Fresno^1,2^

**Materials and Methods**

| **Gene** | **Primer Sequence (5`→3`)** |
| --- | --- |
| TNF-α | F: CCACCACGCTCTTCTGTCTAC  R: AGGGTCTGGGCCATAGAACT |
| IL-1β | F: TGTGAAATGCCACCTTTTGA  R: GGTCAAAGGTTTGGAAGCAG |
| IL-6 | F: TGATGCACTTGCAGAAAACA  R: ACCAGAGGAAATTTTCAATAGGC |
| IL-10 | F: ATCGATTTCTCCCCTGTGAA  R: TGTCAAATTCATTCATGGCCT |
| GAPDH | F: AGGTCGGTGTGAACGGATTTG  R: TGTAGACCATGTAGTTGAGGTCA |
| RPL13A | F: ATCCCTCCACCCTATGACAA  R: GCCCCAGGTAAGCAAACTT |

**Supplementary Table S1**. List of primers used for qPCR.

**FRET image acquisition and processing**

Images of live cells were captured at 60x magnification, under water immersion. Images from three channels were acquired: CFP channel (a CFP excitation and emission filter), a YFP channel (a YFP excitation and emission filter) and a FRET channel (containing a CFP excitation filter and a YFP emission filter). CFP and YFP channel were used to correct the FRET image, because the donor emission can bleed-through into the FRET channel and because CFP excitation wavelength can excite the acceptor YFP, giving false positive FRET signals. Thus, cells expressing only TLR2-CFP or only TLR4-YFP were used as controls. First, images of cells expressing TLR4-YFP were taken, exciting YFP with a 513-laser line and the 458 laser, each time collecting images in the FRET channel (Em 527 nm). Then, images of cells expressing TLR2-CFP were obtained, exciting CFP at 458 nm and acquiring images in both the CFP (Em 480nm) and FRET channel. These images were used to calculate the correction factors A (percentage of YFP in the FRET channel) and B (percentage of CFP in the FRET channel). ROIs were drawn in images of cells expressing only TLR4-YFP as well as in regions without cells to correct for background. The average of intensity values from all acquired YFP images give the correction factor A. The same procedure was performed for images of cells expressing only TLR2-CFP to calculate the correction factor B. Applying the following equation (1)

$FRETCorr=FRETRaw-\left( A*YFP \right)-\left( B*CFP \right)$ (1)

A corrected FRET image (FRETCorr) is obtained from the FRET Raw image of cells co-expressing TLR4-YFP and TLR2-CFP as well as for the CFP:YFP construct. The FRET efficiency is shown as a color-coded scale of values between 0 and 100%. Quantification of the number of FRET positive structures in the cells, as well, as the mean fluorescence intensity of each structure was determined in ImageJ, applying a threshold in grayscale images, to eliminate e4

**Supplementary Figure S1**

**Supplementary Fig. S1** HEK 293/hTLR4A-MD2-CD14 cells were activated with LPS purified from *E. coli* or *O intermedium* at the indicated doses. NF-κB activity was measured 60 min after as percentage of I-κB degraded with respect to control untreated cells

**Supplementary Figure S2**

**
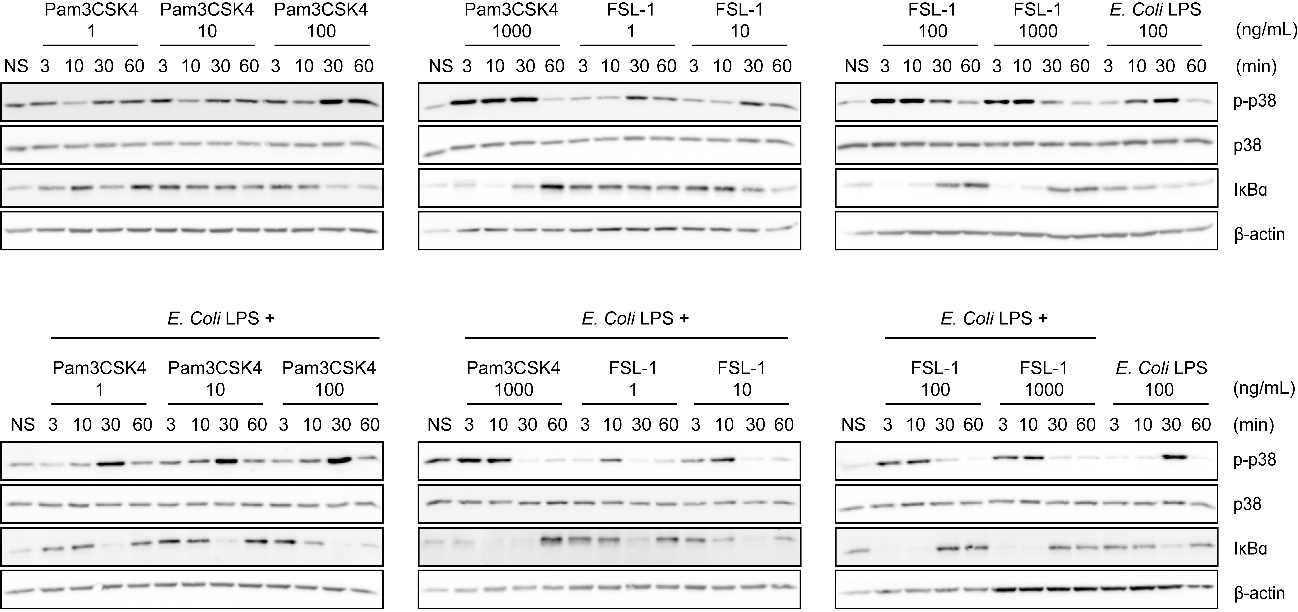
Supplementary Fig. S2 TLR4 and TLR2 co-activation induce an additive effect on NF-κB and p38 pathways.**

Macrophage cell line J774 was left unstimulated (NS) or was stimulated with *E. coli* LPS at 100 ng/mL, *E. coli* LPS plus Pam3CSK4 or *E. coli* LPS plus FSL-1 with the different doses shown in the figure for 3, 10, 30 and 60 min. At each time point, cells were lysed and the cell lysate was subjected to immunoblotting to detect phosphorylated and total p38 MAPK and total IκBα.

**Supplementary Figure S3**

**Supplementary Fig. S3 TLR2 co-activation with Ochrobactrum intermedium LPS induces a much stronger and faster signal on NF-κB pathway.**

Macrophage cell line J774 was left unstimulated (NS) or was stimulated with *Ochrobactrum intermedium* LPS (Oi LPS) at 10 μg/mL alone or plus Pam3CSK4 or FSL-1 with the different doses shown in the figure for 10, 30 and 60 min. At each time point, cells were lysed and the cell lysate was subjected to immunoblotting to detect total IκBα. The data is representative of 2 independent experiments.


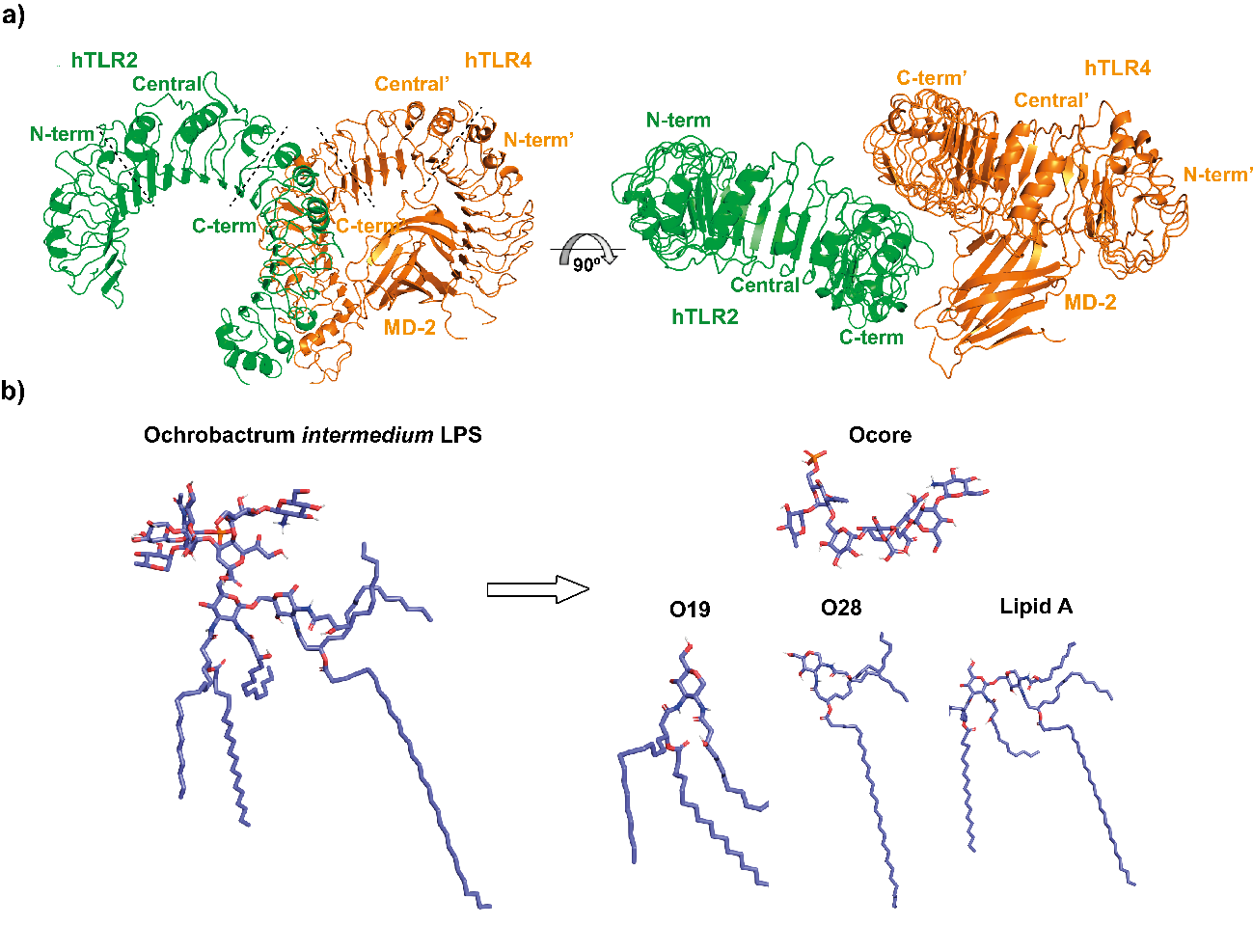
**Supplementary Figure S4**

**Supplementary Fig. S4. hTLR2/TLR4/MD-2 heterodimer model and *O. intermedium* LPS fragmentation**

**a)** 3D structure of hTLR2/TLR4/MD-2 heterodimer model in front view and upper view. TLR2 is represented in green and TLR4 and MD-2 in orange. **b)** Representation of the full *O. intermedium* LPS structure and the obtained fragments: the core saccharide (Ocore), the fragment with the two fatty acid chains containing 12 and 16 carbons and the third 28 carbon acyl chain attached to C16 (O28), the fragment containing the two lipid chains with 14 and 18 carbons and the third 19 carbon lipid chain attached to C18 (O19), and the lipid A.


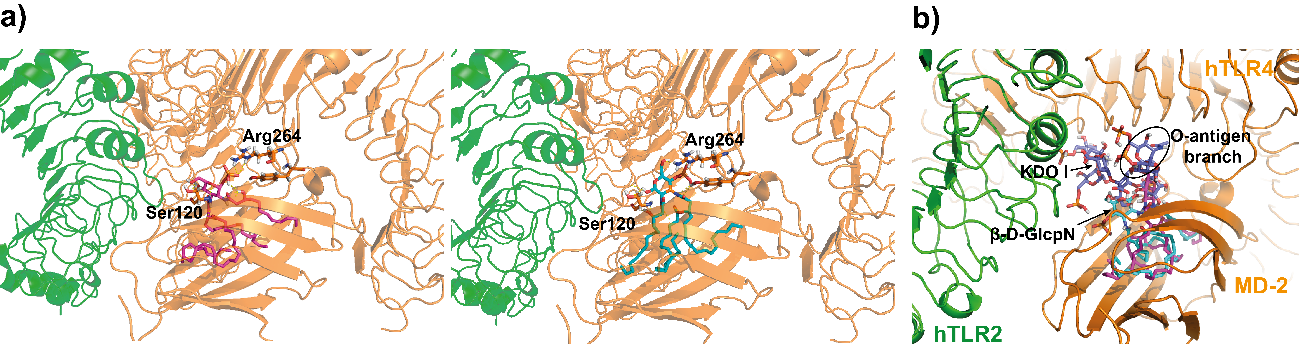
**Supplementary Figure S5**

**Supplementary Fig. S5. View of the fragments O28, O19 and Ocore docked in the hTLR4/MD-2 pocket.**

**a)** O28 (in magenta) and O19 (in cyan) docked in the TLR4/MD-2 pocket. **b)** Superimposition of the three fragments (Ocore in purple) docked in MD-2 pocket (TLR4 and MD-2 are displayed in orange and TLR2 in green).

**Supplementary Figure S6**


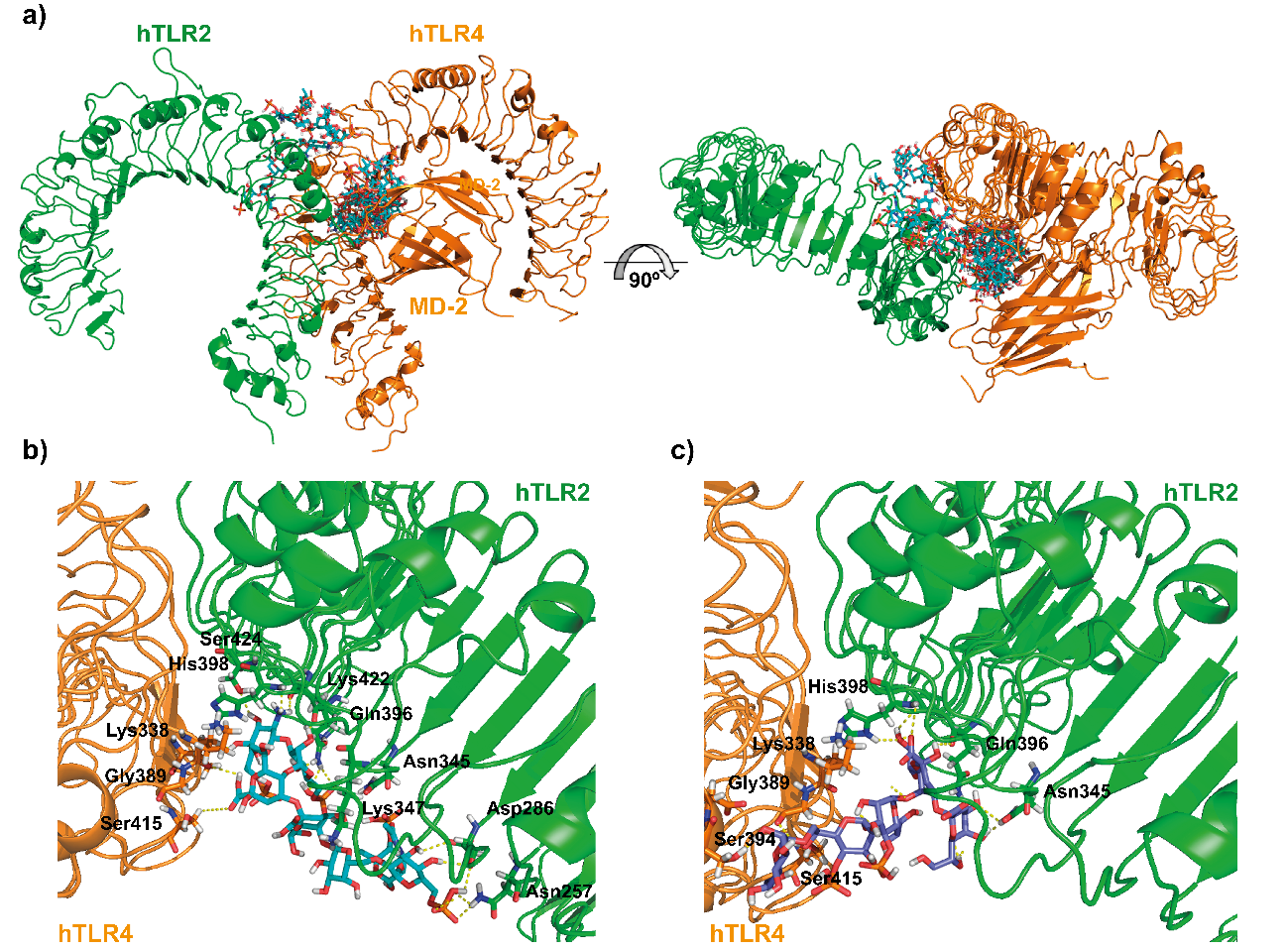


**Supplementary Fig. S6.** **Docking of *O. intermedium* LPS core in hTLR2 central and C-terminal domain in the hTLR2/TLR4/MD-2 complex.**

Front and top view of Ocore fragment (in cyan) docked poses in hTLR2 (in green) and hTLR4/MD-2 (in orange) interface. **b)** Hydrogen interactions of Ocore fragment with hTLR2 (green sticks) and hTLR4 residues (orange sticks). **c)** Hydrogen bonds of *E. coli* core fragment with hTLR2 (green sticks) and hTLR4 residues (orange sticks).

**Supplementary Figure S7**

**Supplementary Fig. S7.** **Structures of *O. intermedium* and *E. coli* LPSs**

Mayor differences are in the much longer length of one acyl chain of the lipid A and in the core structure.
